# Astrocytic GPCR-Induced Ca^2+^ Signaling Is Not Causally Related to Local Cerebral Blood Flow Changes

**DOI:** 10.3390/ijms241713590

**Published:** 2023-09-02

**Authors:** Katsuya Ozawa, Masaki Nagao, Ayumu Konno, Youichi Iwai, Marta Vittani, Peter Kusk, Tsuneko Mishima, Hirokazu Hirai, Maiken Nedergaard, Hajime Hirase

**Affiliations:** 1 Laboratory for Neuron-Glia Circuitry, RIKEN Center for Brain Science, Wako 351-0106, Saitama, Japan; katsuya.ozawa@riken.jp (K.O.);; 2 Center for Translational Neuromedicine, Faculty of Medical and Health Sciences, University of Copenhagen, 1172 Copenhagen, Denmark; 3 Department of Neurophysiology and Neural Repair, Gunma University Graduate School of Medicine, Maebashi 371-8511, Gunma, Japan; 4 Viral Vector Core, Gunma University, Initiative for Advanced Research, Maebashi 371-8511, Gunma, Japan; 5 Center for Translational Neuromedicine, University of Rochester Medical Center, Rochester, NY 14642, USA

**Keywords:** astrocytes, optogenetic GPCR, OptoA1AR, Ca^2+^ elevation, blood flow, cortex, mouse, optical imaging

## Abstract

Activation of Gq-type G protein-coupled receptors (GPCRs) gives rise to large cytosolic Ca^2+^ elevations in astrocytes. Previous in vitro and in vivo studies have indicated that astrocytic Ca^2+^ elevations are closely associated with diameter changes in the nearby blood vessels, which astrocytes enwrap with their endfeet. However, the causal relationship between astrocytic Ca^2+^ elevations and blood vessel diameter changes has been questioned, as mice with diminished astrocytic Ca^2+^ signaling show normal sensory hyperemia. We addressed this controversy by imaging cortical vasculature while optogenetically elevating astrocyte Ca^2+^ in a novel transgenic mouse line, expressing Opto-Gq-type GPCR Optoα1AR (Astro-Optoα1AR) in astrocytes. Blue light illumination on the surface of the somatosensory cortex induced Ca^2+^ elevations in cortical astrocytes and their endfeet in mice under anesthesia. Blood vessel diameter did not change significantly with Optoα1AR-induced Ca^2+^ elevations in astrocytes, while it was increased by forelimb stimulation. Next, we labeled blood plasma with red fluorescence using AAV8-P3-Alb-mScarlet in Astro-Optoα1AR mice. We were able to identify arterioles that display diameter changes in superficial areas of the somatosensory cortex through the thinned skull. Photo-stimulation of astrocytes in the cortical area did not result in noticeable changes in the arteriole diameters compared with their background strain C57BL/6. Together, compelling evidence for astrocytic Gq pathway-induced vasodiameter changes was not observed. Our results support the notion that short-term (<10 s) hyperemia is not mediated by GPCR-induced astrocytic Ca^2+^ signaling.

## 1. Introduction

Functional hyperemia is a process to provide an on-demand metabolic supply, whereby areas of high neural activity receive higher blood flow by dilation of local blood vessels [1]. Understanding functional hyperemia is important since brain activity imaging techniques, including functional magnetic resonance imaging (fMRI), rely on activity-dependent cerebral blood flow changes. Several mechanisms have been proposed, such as neuronal activity-triggered increases in nitric oxide, prostaglandins, adenosine, and potassium [2,3,4,5].

Astrocytes are a major glial cell type that extends their processes to neighboring blood vessels and synapses via endfeet and peri-synaptic astrocytic processes, respectively. In the cerebral cortex gray matter, astrocytes represent a significant portion of the cellular composition, ranging from 20–30% in rodents and humans [6,7,8,9]. Astrocytes express receptors for neurotransmitters and neuromodulators, most of which are G protein-coupled receptors (GPCRs) [10]. Large-amplitude cytosolic Ca^2+^ elevations occur in astrocytes by Gq-coupled GPCRs, which activate the inositol trisphosphate (IP_3_) pathway. 

Astrocytes, with their extensive blood vessel coverage and ability to sense neuronal activity, represent an optimal candidate cell type for mediating functional hyperemia. Indeed, astrocytic Ca^2+^ elevation has been implicated in the modulation of local cerebral blood flow by multiple independent groups [11,12,13,14]. However, this role of astrocytic Ca^2+^ has been questioned since IP_3_ receptor type-2 knockout mice (IP_3_R2-KO), in which large astrocytic Ca^2+^ elevations are diminished, display a similar extent of functional hyperemia [15,16,17]. The controversy remains unresolved to date, as a residual Ca^2+^ response was observed in IP_3_R2-KO astrocytes, including their endfeet [18,19], though vastly reduced in amplitude [20]. Moreover, other studies reported a tight relationship between astrocyte endfoot Ca^2+^ and vessel diameter or blood flow velocity [21,22], and proposed an indirect astrocyte–capillary vasomodulation via pericytes [23]. Utilization of genetic cytosolic Ca^2+^ indicators, suitable for detecting GPCR-induced Ca^2+^ increases from internal stores, did not support astrocytic modulation of vessel changes [24,25], whereas use of genetic membrane-tethered Ca^2+^ indicators for detection of endfoot Ca^2+^ events [19] gave mixed results [20,26].

The lack of a hypothesized phenotype by genetic loss-of-function approaches could be due to a compensatory response in the system. A gain-of-function approach that elevates cytosolic Ca^2+^ selectively in astrocytes can test the causal involvement of astrocytic Ca^2+^ in vasodiameter regulation. Indeed, Bonder and McCarthy (2014 [15]) demonstrated that activation of astrocytes by a pharmacogenetic Gq-GPCR (hM3Dq DREADD) did not lead to hyperemia. Still, DREADD-induced Ca^2+^ signaling is complex [27], and the Ca^2+^ dynamics may differ from physiologically induced Ca^2+^ patterns. Here, we chose optogenetics as the least invasive method for astrocyte manipulation. We used a transgenic mouse line expressing the optogenetically activated Gq-type GPCR Optoα1AR in astrocytes, where astrocytic Ca^2+^ elevation is induced by brief blue light illumination (Astro-Optoα1AR, BAC-GLT1-Optoα1AR-EYFP #941; [28,29]). Forelimb stimulation in anesthetized mice resulted in reliable blood vessel dilations in the corresponding somatosensory cortex. However, Optoα1AR-induced astrocyte-selective Ca^2+^ elevation did not lead to vessel diameter changes in the same set of vessels that showed functional hyperemia. Furthermore, AAV-mediated labeling of blood plasma [30] in Astro-Optoα1AR mice allowed for non-invasive monitoring of cerebral arterioles via the thinned skull in awake mice. Yet, optogenetic activation of the astrocytic Gq pathway did not impact the spontaneous vessel diameter changes in these arterioles.

## 2. Results

In order to elevate Ca^2+^ in astrocytes via the IP_3_ pathway at a given time and location, we used a transgenic mouse line in which Optoα1AR is expressed in astrocytes under the control of a BAC-GLT1 promoter (Astro-Optoα1AR; BAC-GLT1-OptoA1AR-EYFP #941, [28]). Since repeated in vivo activation of Optoα1AR requires cis-retinal supplement [28], mice were injected with 9-cis-retinal intraperitoneally 20–30 min before imaging.

**Figure 1:**
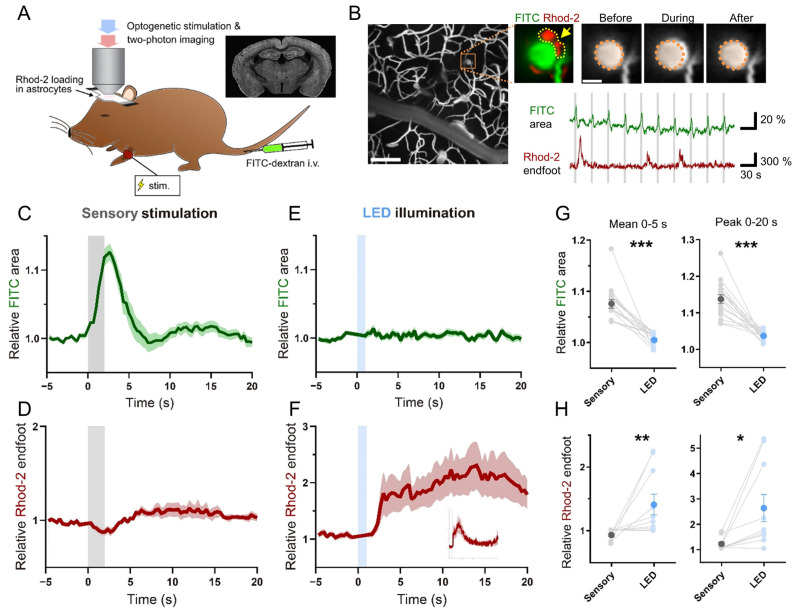
Transient astrocytic Optoα1AR activation did not induce artery dilation in vivo. (**A**) Two-photon vasculature imaging from layer 2/3 somatosensory cortex of urethane-anesthetized Astro-Optoα1AR mice upon LED illumination. Blood vessels were labeled by i.v. injection of FITC-dextran and astrocytes were loaded with the Rhod-2 Ca^2+^ indicator. LED illumination (1 mW, 1 s) or sensory stimulation (duration 2 s, interval 30 s) were delivered through the objective lens or on the forelimb, respectively. Inset: a representative coronal brain section showing EYFP fluorescence that is part of the Optoα1AR transgene construct. (**B**) The low-power view shows the pattern of FITC-filled blood vessels. The high-power view of the orange rectangle region contains a FITC-filled penetrating arterial cross-section (green) and a Rhod-2-loaded astrocytic endfoot (red), Region of interest (ROI) of the endfoot is the yellow semi-lucent area indicated by the arrow. The artery expanded during the sensory stimulation. The semi-lucent orange area represents the arterial area before sensory stimulation. The arterial area expanded reliably at every sensory presentation, whereas the endfoot Ca^2+^ signal increased only occasionally. Scale bars: 100 μm (low-power image); 10 μm (high-power image); 20% (FITC area trace), 300% (Rhod-2 F/F0 trace), 30 s (time axis). (**C**) Arterial cross-sectional area rapidly increased immediately after the sensory stimulation onset (17 arterial cross-sections from 5 strong TG mice). SEM is shown by the shaded region. (**D**) Averaged endfoot Ca^2+^ signal shows marginal changes after the sensory stimulation (10 endfeet from 3 strong TG mice). (**E**) Arterial cross-sectional area of the same data set as in (**C**) did not increase upon the LED illumination. (**F**) Endfoot Ca^2+^ signal of the same dataset as in (**D**) strongly increased after the LED illumination. Insets: Endfoot Ca^2+^ change by LED illumination is shown in full on an expanded time scale. Abscissa: −15 s to 90 s, where time 0 corresponds to the onset of LED illumination. Ordinate: −0.5 to 3.0 F/F0. (**G**) Mean and peak values of arterial cross-sectional area are plotted for the periods 0–5 s and 0–20 s (time 0 is the onset of sensory stimulation). Both measures are significantly larger than those of LED illumination (*** *p* < 0.001, paired *t*-test). (**H**) Mean and peak values of endfoot Ca^2+^ signals are plotted for the periods 0–5 s and 0–20 s (time 0 is the onset of LED illumination). Both measures are significantly higher than those of sensory stimulation (* *p* < 0.05, ** *p* < 0.01, paired *t*-test).

To test whether astrocytic Gq signaling has a causal impact on local cerebral blood flow, we performed two-photon imaging of the vasculature in the somatosensory cortex of lightly urethane-anesthetized mice (Figure 1A). The vasculature was labeled by i.v. FITC injection, and astrocytes were loaded with an organic Ca^2+^ indicator Rhod-2 AM. First, we confirmed that brief forelimb stimulation (2 s) induces dilation of selected penetrating arterioles in the primary somatosensory cortex forelimb area. The arteriole cross-section expanded immediately after stimulus onset (<1 s) and peaked in three seconds. Thereafter, the arteriole cross-sectional area gradually decreased to baseline in five seconds. This response was stereotypical and repeatable across multiple presentations of forelimb stimulation at an interval of 30 seconds (Figure 1B). The degree of arteriole expansion did not significantly differ between the first three and the last three trials of the nine consecutive trials (*p* > 0.7, paired t-test). Overall, the relative area increase in arterioles in the first 3 s after stimulus onset was 12.6 ± 1.1% (17 arterioles, 5 mice, Figure 1C and Figure S1, Supplementary Data S1). On the other hand, arteriole area increase in the hindlimb somatosensory cortex was negligible (1.9 ± 0.6%, 4 arterioles, 2 mice), suggesting that the arteriole dilation was locally induced. Astrocytic endfoot Ca^2+^ elevations were only occasionally observed after sensory stimulation (occurrence probability = 10/100 = 10%; 10 endfeet, 3 mice). On average, endfoot Ca^2+^ increase (ΔF/F0) of 12 ± 5% was observed in the averaged plot with a latency of several seconds (Figure 1D).

Having demonstrated reliable functional hyperemia, we examined if optically evoked Gq-induced astrocytic Ca^2+^ elevation impacts the vasomodulation of the same set of arterioles. As in Figure 1A, a brief illumination of blue light (1 s, 1 mW) through the objective lens was given while arteriole cross-sections and astrocytic Ca^2+^ were imaged. We observed that arteriole cross-sectional areas remained unchanged despite optical stimulation (Figure 1E, Supplementary Data S1), while Ca^2+^ elevations at astrocytic endfeet were reliably induced (Figure 1F). The lack of arteriole cross-section change was in stark contrast with sensory-induced hyperemia (Figure 1G), despite significant Ca^2+^ increases in the astrocytic endfeet by optogenetic Gq-GPCR stimulation (Figure 1H and Figure S2). Taken together, these experiments dissociated the role of astrocytic Ca^2+^ elevations from sensory-driven arteriole dilation.

**Figure 2:**
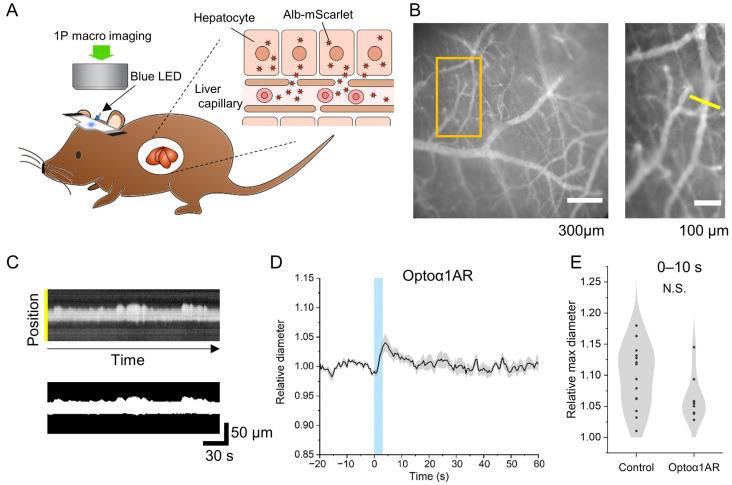
Vasculature imaging and activation of astrocytic Optoα1AR in awake mouse cortex through the thinned skull. (**A**) Fluorescence imaging of cortical vasculature and optogenetic activation of astrocytes in awake mice. The somatosensory cortex was imaged. Optoα1AR and control mice have received liver-targeting AAV8-P3-Alb-mScarlet injection for red fluorescent blood plasma labeling. Optogenetic stimulation is provided by a blue LED (465 nm, ~3 mW, 3 s), targeted at the thinned skull area. (**B**) Macroscopic imaging of Alb-mScarlet-labeled cerebral vasculature. In the example, the orange box area in the left panel is magnified in the right panel, and the vessel section by the yellow line is identified to be an arteriole, as the line profile through time (**C**) shows dynamic fluctuations of the vessel diameter (top: original line profile, bottom: thresholded binary line profile). (**D**) Normalized dynamics of cerebral arteriole diameter dynamics in Astro-Optoα1AR mice. The blue shade indicates the period of optogenetic stimulation. Modest dilation is observed within several seconds after the onset of optogenetic stimulation. (**E**) Comparison of transient arteriole diameter changes induced by blue LED cortical illumination. The maximum diameter recorded during 0–10 s after the onset of optogenetic stimulation is compared. Both control and Astro-Optoα1AR mice display significant increases (*p* < 0.001), while the increase is not significantly (N.S.) different between control and Astro-Optoα1AR mice (*n* = 14 vs. 9, *p* = 0.07).

Next, we investigated possible vasomodulatory effects of wider-area astrocytic Gq-GPCR activation in awake mice. For chronic visualization of blood plasma by red fluorescence, we injected AAV8-P3-Alb-mScarlet retro-orbitally to Astro-Optoα1AR and C57BL/6 control mice 4–5 weeks before the imaging experiment, which allowed for imaging of superficial vessels by fluorescence microscopy [30]. The vasculature in the somatosensory area was examined through the thinned and wetted skull using a fluorescence macroscope. Red fluorescence in pial vessels and their continuation into a shallow depth in the parenchyma were visualized (Figure 2A,B). Arterioles were recognized by their morphology (branching pattern and diameter) and spontaneous diameter dynamics that occurred in 10 minutes (Figure 2C). To examine the effect of astrocytic Ca^2+^ elevation, the thinned skull area was illuminated with a blue LED for 3 s (center wavelength: 465 nm, 3 mW, ~2 × 2 mm^2^), which was shown to be sufficient to induce large cytosolic Ca^2+^ elevations in Astro-Optoα1AR mice [28] after light scattering and attenuation by the skull [31]. The arteriole diameter profiles of Astro-Optoα1AR mice showed a transient increase lasting for 10 seconds (Figure 2D, % max diameter during 0–10 s since LED onset: 14.5 ± 1.2%, 9 arterioles from 5 mice, Supplementary Data S1). However, control mice also showed arteriole diameter increases (17.9 ± 1.2%, 14 arterioles from 5 mice) in response to cortical surface LED illumination, which were similar to Optoα1AR mice (Figure 2E, Astro-Optoα1AR vs. control, t = 1.9, *p* = 0.07). Hence, the vascular response is conceivably due to visual sensing of the LED cortical illumination by the conscious mice, which in turn induced large-scale startle-related brain activity.

**Figure 3:**
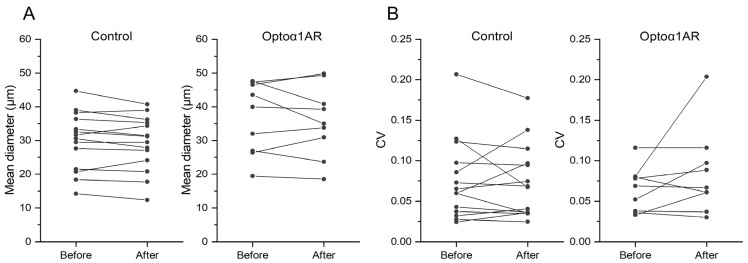
Intermittent astrocytic Optoα1AR activation did not induce obvious effects on cortical arteriole diameter or vasodynamics. Arteriole diameter dynamics were monitored for 10 min before and after the optogenetic experiment presented in Figure 2. (**A**) Mean diameters for the respective 10 min period are plotted for control (left) and Astro-Optoα1AR (right). No significant difference was observed in either group (Ctrl: *p* = 0.18, Astro-Optoα1AR: *p* = 0.53) (**B**) Coefficients of variation for arteriole diameter profile for the respective 10 min period are plotted. No significant difference was observed in either group (Ctrl: *p* = 0.82, Astro-Optoα1AR: *p* = 0.20).

Finally, we examined the effects of the repeated optogenetic stimulation (6 × 3 s, ~3 mW in 20 min) on vessel diameter and spontaneous vasomotion by imaging the vasculature for 10 min before and after the optogenetic stimulation experiment. The mean diameter (Figure 3A) and coefficient of variation (CV, Figure 3B) computed over 10 min were similar between, before, and after the optogenetic stimulation experiment for both control (mean diameter: *p* = 0.18, CV: *p* = 0.82) and Astro-Optoα1AR (mean diameter: *p* = 0.53, CV: *p* = 0.20) mice. These results collectively indicated that the intermittent activation of Gq-induced astrocytic Ca^2+^ elevation does not make a visible impact on minute-scale vasodynamics.

## 3. Discussion

The involvement of astrocytic Ca^2+^ signaling in functional hyperemia has been controversial [32,33,34,35,36,37,38], in part due to the lack of astrocyte-selective manipulation. Negative results from in vivo experiments in IP_3_R2-KO mice [15,16,17,39] have cast doubt on previous work using pharmacological interventions and/or in vitro work, though residual small-scale Ca^2+^ signals are observable in IP_3_R2-KO astrocytic processes [18,40,41]. Astrocytic microprocesses have been reported to show sub-second responses to sensory-evoked neuronal activity (most likely synaptic transmission) [22,41], on a time scale faster than hemodynamical responses, and some studies have suggested active role of astrocyte endfoot fast Ca^2+^ signaling in sensory evoked vasomodulation [21,22,26], though without causal manipulations. On the other hand, a recent study using IP_3_R2-KO concluded that there was no causal role of astrocytic Ca^2+^ signaling in cerebral cortical capillaries [20].

Optogenetic astrocyte stimulation with channelrhodopsin-2 (ChR2) has been performed in the context of cerebral blood flow modulation using a transgenic mouse line, allowing for non-invasive astrocyte-selective activation [42]. The study reported a widespread cerebral cortical blood flow increase stemming from the optogenetic stimulation site that propagated to the contralateral side. Similar increases in fMRI BOLD signals have also been reported [43]. Considering extracellular increase in potassium after astrocytic ChR2 stimulation [44], astrocytic ChR2 activation conceivably resulted in elevation of neuronal excitability and/or energy metabolism and induced hyperemia. Moreover, since astrocytes do not have notable active membrane conductance or proton channels, ChR2 activation may not mimic physiological mechanisms of functional hyperemia. Likewise, rapid Ca^2+^ increase by photolysis of caged Ca^2+^ has been reported to induce a differential biochemical pathway from that induced by GPCRs [45].

The current study leverages an optogenetic GPCR that mimics the in vivo astrocytic Ca^2+^ dynamics induced by pronounced sensory input [28], while channel-mediated, shorter, and smaller Ca^2+^ signals [46] remain unaddressed. We confirmed that sensory stimulation causes occasional astrocytic Ca^2+^ elevations [41,47,48]. Such stimulation caused reliable functional hyperemia, agreeing with a previous paper that astrocytic Ca^2+^ events are not necessary for functional hyperemia [47]. Furthermore, we made a more refined observation that astrocytic endfoot Ca^2+^ events are not necessary for functional hyperemia in the somatosensory cortex by two-photon microscopy, confirming a previous study in the visual cortex [15]. Vasodynamics has been proposed to be differentially affected depending on the level of astrocytic Ca^2+^ [49]. The current work assessed a strong Ca^2+^ elevation pattern that is within the physiological range; hence, more subtle Ca^2+^ signaling requires further study.

Although pharmacogenetic approaches with systemic injection of ligands activate the system for tens of minutes, the Astro-Optoα1AR mouse allows for triggering transient astrocytic Ca^2+^ elevations with the precision of a second with the Gq-pathway-induced dynamics [28,29]. Inducing astrocytic Ca^2+^ elevations in Astro-Optoα1AR mice, however, did not result in penetrating arteriole dilations in the same set of arterioles that displayed sensory hyperemia. Cortical hyperemia has been demonstrated to be actuated by smooth muscle cells [50] and/or some capillary pericytes whose activation precedes that of upstream arterioles [51]. Since we did not examine capillary diameters, astrocyte endfoot Ca^2+^ elevation remains a viable candidate pathway for microcirculatory functional hyperemia. Of note, a recent study using IP_3_R2-KO mice reported little causal involvement of endfoot Ca^2+^ elevation in capillary expansion in the cerebral cortex [20]. The reported neuronally evoked capillary dilatation may still be controlled by endfoot Ca^2+^ entry [22], possibly via P2X channels [23], while astrocyte-specific interference experiments would substantiate this mechanism. Interestingly, a recent study that overexpressed Ca^2+^ extrusion pump (CalEx) in somatosensory cortex astrocytes reported normal hyperemia in response to 5 s whisker stimulation, whereas prolonged stimulation (30 s) resulted in a mild enhancement in arteriole dilation [52]. Considering that the Optoα1AR activation in our study results in ~30 s astrocytic Ca^2+^ elevation and little arteriole dilation, it is possible that prolonged astrocytic Ca^2+^ elevations have an impact on the modulation of arteriole dilation.

There is a concern that a few milliwatts of fiber-guided blue light induces a positive hemodynamic response in ketamine–xylazine-anesthetized mice [53]. While the stimulation light power was 3 mW for our experiment with awake mice, the illumination was over a larger area on the thinned skull, which is considered to have a lower per-volume intensity (angled 62.5 µm core fiber-delivered illumination to the cranial window vs. 2 × 2 mm^2^ area). Nonetheless, the direct effect of blue light cannot be excluded from the mechanisms contributing to the small hyperemia seen in both Optoα1AR and C57BL/6 mice.

To conclude, our results with the refined and non-invasive temporal actuation of astrocytic Ca^2+^ elevation lend support to the notion that Gq-GPCR-induced large-amplitude and long-lasting astrocytic Ca^2+^ surge is not sufficient to induce functional hyperemia, in agreement with Bonder and McCarthy (2014 [15]).

## 4. Materials and Methods

### 4.1. Mice

Adult male and female heterozygous Astro-Optoα1AR mice (>2 months old, BAC-GLT1-OptoA1AR-EYFP #941, Jackson Laboratory strain ID: 038174) [29] and age-matched background strain mice (C57BL/6J) were used in this study. All experimental procedures involving mice were approved by the RIKEN Institutional Animal Care and Use Committee of the Danish Animal Experiments Inspectorate.

### 4.2. Surgery for Acute In Vivo Two-Photon Imaging

Adult Astro-Optoα1AR mice were anesthetized (1.0 g/kg urethane and 50 mg/kg α-chloralose). A metal frame was attached to the skull of each anesthetized mouse using dental acrylic (Fuji LUTE BC, GC, Tokyo, Japan and Super Bond, Sun Medical, Shiga, Japan). A small craniotomy was made above the primary somatosensory cortex (diameter 2.0–3.0 mm AP −1.0 to −2.0 mm and ML 1.5 to 3.5 mm). The dura mater was carefully removed, and the exposed cortex was loaded with Rhod-2 AM (0.4 mM, Molecular Probes-Invitrogen, Eugene, OR, USA) for 1 h. After washing with HEPES-ACSF (125 mM NaCl, 3.5 mM KCl, 10 mM glucose, 10 mM HEPES, 2 mM CaCl_2_ and 2 mM MgSO_4_, pH 7.3) several times, the craniotomy was sealed with a thin glass coverslip.

### 4.3. In Vivo Two-Photon Imaging

A two-photon microscope (Bergamo, Thorlabs, Newton, NJ, USA) with a Chameleon Ultra 2 laser (Coherent, Santa Clara, CA, USA), a 25× objective lens (1.05 NA, Olympus, Tokyo, Japan), a primary dichroic mirror (405/473–488/561/705–1600 nm notched dichroic mirror, Thorlabs), and a secondary dichroic mirror cube for emission light (FF562-Di03, FF03-525/50, FF01-607/70, all from Semrock, New York, NY, USA) was used, as described previously [28]. Images were acquired using the proprietary software ThorImage LS v3.0. Simultaneous imaging of astrocytic endfoot Ca^2+^ (Rhod-2) and the vasculature (FITC) was performed using a mode-locked beam of 820 nm light. Photo-activation of Optoα1AR was carried out with a 470 nm LED (M470L3, Thorlabs) targeted at the imaged area (φ = ~0.8 mm) through the objective lens. The photomultiplier tubes (PMTs) were blocked by a built-in mechanized shutter during photo-activation.

On the day of the experiment, an aliquot of 9-cis-retinal (5 µL, 200 mM in dimethyl sulfoxide; R5754, Sigma-Aldrich, St. Louis, MO, USA) was diluted in 100 µL HEPES-ACSF or saline at 35 °C and administered by intraperitoneal (i.p.) injection ~30 min before the imaging session. Thereafter, an intravenous injection of FITC-dextran was made to label the serum via the femoral vein (50–70 µL, 5% in saline; FD-2000S, Sigma-Aldrich), similar to the previously described procedure [54]. After mounting the mouse under the objective lens via the headplate, needle electrodes (30 G) were inserted in the contralateral forelimb to apply sensory stimulation (1 mA, 100 ms, 3 Hz, 6 times, interval 30 s, 10 times). Imaging was performed at a frame rate of 3 Hz. Once sensory-driven vasodilation was observed, the same area was imaged with Optoα1AR activation by LED illumination (1 mW, duration 1 s). In some mice (3 out of 5 mice), astrocytic Rhod-2 loading in the somatosensory cortex was also done. For analysis of FITC area, penetrating arterioles that had circular cross-sections were sampled.

### 4.4. Imaging of Awake Mice

Adult Astro-Optoα1AR and control mice were briefly anesthetized by isoflurane (3%), and retro-orbital administration of AAV8-P3-Alb-mScarlet (2–4 × 10^11^ vg in ~150 µL saline; Addgene 183461) was made at least four weeks prior to imaging [30]. The headplate attachment was made as described above. The skull was thinned by a dental drill to the extent that dural vessels are observable through the saline-wet thinned skull.

Next, 9-cis-retinal was administered by i.p. injection ~20 min before the imaging session, as described above. Awake mice were fixed under a macroscope (Leica M205 FA, Leica, Wetzlar, Germany) equipped with an X-Cite 200Dc light source and a digital camera (ORCA-Flash4.0, Hamamatsu, Shizuoka, Japan). Cerebral vessels of the thinned skull area were observed using an ET mCherry filter set (excitation 560/40 nm, emission 630/75 nm; 10450195, Leica). For optogenetic stimulation, a blue LED (center wavelength: 465 nm; NSPB300B, Nichia, Tokushima, Japan) was mounted onto the microscope apparatus to illuminate the thin-skulled area at an intensity of ~ 3 mW.

The cerebral vasculature through the thinned skull area was imaged at a frame rate of 16.6 Hz (512 × 512 pixels) or 5.2 Hz (1024 × 1024 pixels). First, the vasculature was imaged for 10 min without optogenetic stimulation. Thereafter, six consecutive optogenetic stimulation sessions were recorded, each session consisting of a 20 s pre-stimulation, a 3 s optogenetic stimulation, and a 180 s post-stimulation period. Finally, another 10 min recording was made without optogenetic stimulation.

### 4.5. Data Analysis and Statistics

The analysis for two-photon imaging was performed using ImageJ (ver. 1.45–1.54e; NIH, Bethesda, MD, USA)and MATLAB (R2016a–R2022a; MathWorks, Natick, MA, USA) software. Image shift in the XY axis was adjusted by the TurboReg ImageJ plug-in (7th July, 2011) program for all images. FITC signals were binarized and circular cross-sections of penetrating arterioles were extracted using ImageJ. The binarized data were used to calculate the cross-sectional area using MATLAB. Astrocytic endfeet areas were manually marked in the area surrounding chosen penetrating arterioles and Rhod-2 signals were extracted using ImageJ. The Rhod-2 signals were converted to F/F_0_ using MATLAB. The values of FITC and Rhod-2 were averaged across imaging trials and normalized to the control period of 0–10 s before sensory stimulation or LED illumination. The mean ± SEM of the normalized trace is presented in Figure 1C–F. For statistical comparison of arteriole cross-section, the mean and the peak values of normalized FITC signals from 0 to 5 s and 0 to 20 s after the onset of sensory or LED stimulation were computed (Figure 1G). For comparison of endfoot Ca^2+^, the mean values of normalized Rhod-2 signals from 0 to 20 s after stimulation were computed (Figure 1H).

For macroscopic fluorescence imaging, the original image series were resampled to 2 Hz to enhance pixel intensity range and registered for XY shifts using TurboReg ImageJ plug-in. Horizontally vascularized arterioles were identified by their morphological characteristics, lateral origin (where traceable), and spontaneous vasodiameter changes observed during the 10 min imaging recorded prior to an optogenetic stimulation session. The image stack was binarized by a manually determined threshold that captured a selected arteriole. The arteriole diameter was estimated by drawing an orthogonal line and quantifying the number of positive pixels in the pixel intensity profile. Temporal arteriole diameter profiles were normalized to the mean of each arteriole.

Descriptive statistical values are described as mean ± SEM (standard error of the mean). Comparisons between two groups were analyzed by unpaired *t*-test. If variances were significantly different between them, Welch’s correction was applied (Welch’s *t*-test). For comparisons of data before and after manipulation, paired *t*-test was used.

## Data Availability

Raw data are attached as supplemental materials.

## Supplementary Materials

The supporting information can be downloaded at: https://www.mdpi.com/article/10.3390/ijms241713590/s1.
