# Supplementary figures and images for: Astrocytic GPCR-Induced Ca2+ Signaling Is Not Causally Related to Local Cerebral Blood Flow Changes
Source: Int J Mol Sci. 2023 Sep 2;24(17):13590. doi: 10.3390/ijms241713590 (PMC10487464; doi:10.3390/ijms241713590)

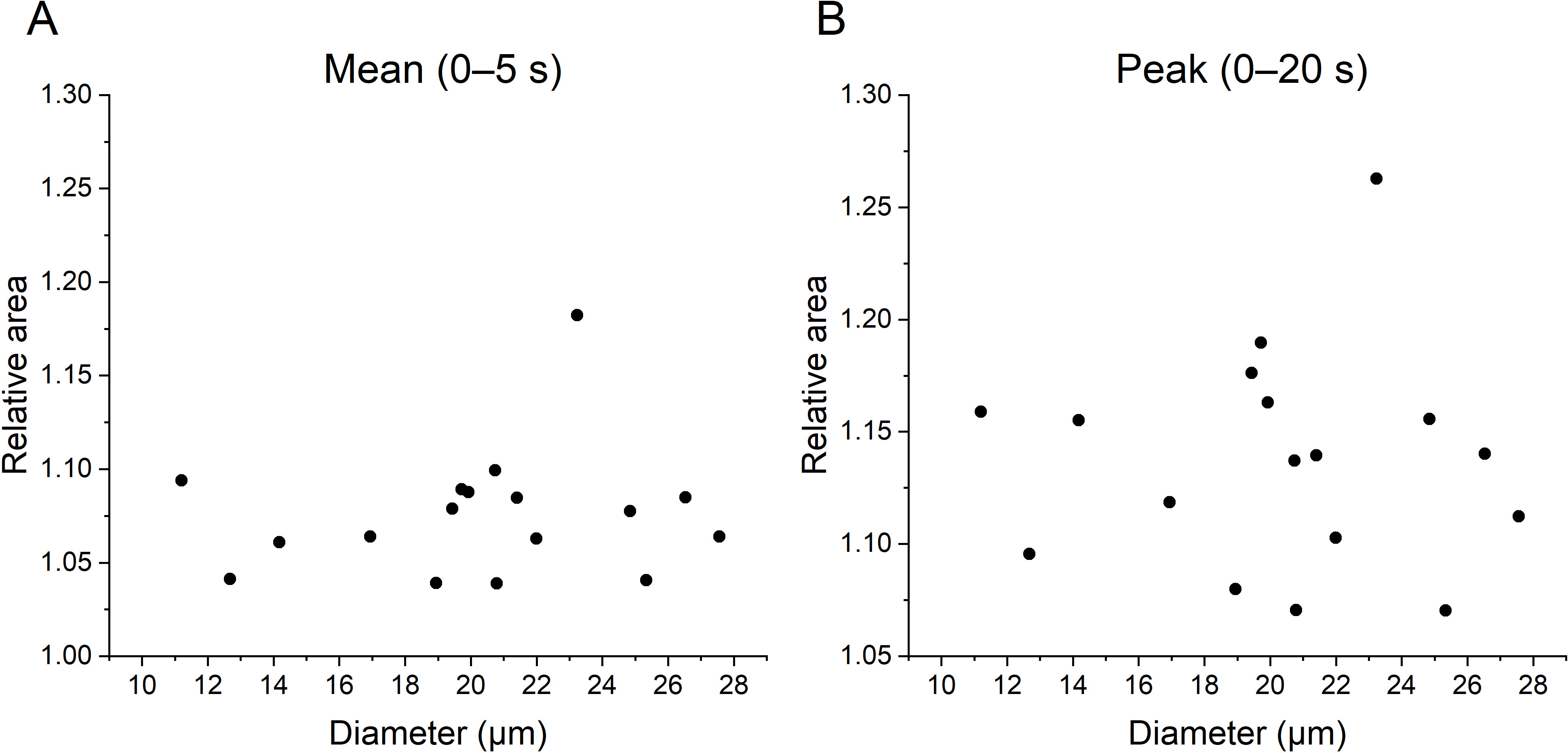

Supplement: Supplementary file 1 [file ijms-24-13590-s001.zip › Figure S1.jpg]

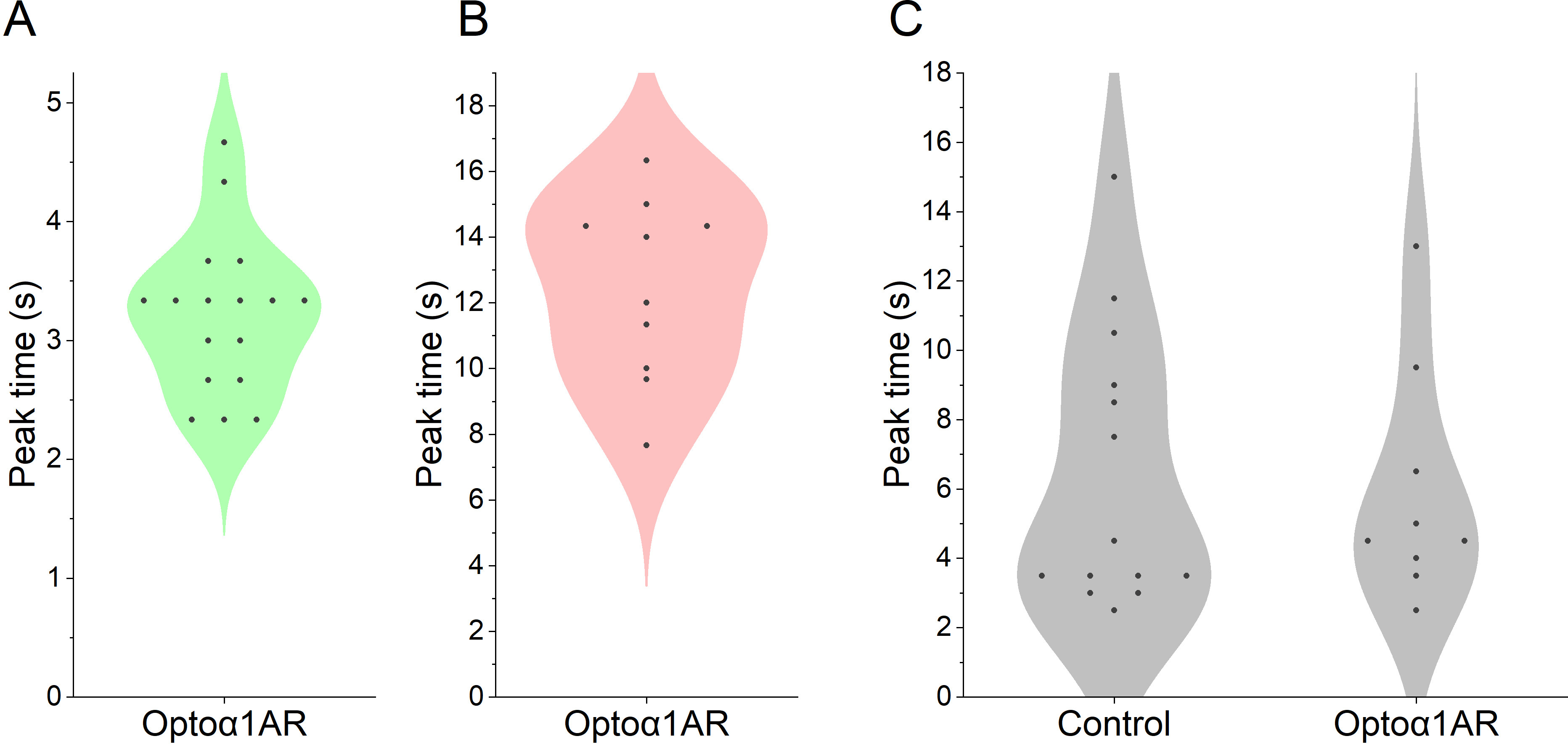

Supplement: Supplementary file 1 [file ijms-24-13590-s001.zip › Figure S2.jpg]
